# Aboriginal medical services cure more than illness: a qualitative study of how Indigenous services address the health impacts of discrimination in Brisbane communities

**DOI:** 10.1186/1475-9276-13-56

**Published:** 2014-10-10

**Authors:** Josifini T Baba, Claire E Brolan, Peter S Hill

**Affiliations:** School of Population Health, Faculty of Medicine and Biomedical Science, The University of Queensland, Queensland, Australia

**Keywords:** Discrimination, Indigenous, Aboriginal, Community controlled health services, Healthcare-seeking behaviour, Fear, Mental health, Australia

## Abstract

**Background:**

Aboriginal and Torres Strait Islanders persistently experience a significantly lower standard of health in comparison to non-Indigenous Australians. The factors contributing to this disparity are complex and entrenched in a history of social inequality, disempowerment, poverty, dispossession and discrimination. Aboriginal medical services (AMS) provide a culturally appropriate alternative to mainstream medical services as a means to address this health disparity and also advocate for Indigenous rights and empowerment. This study provides a vignette of lay perspectives of Aboriginal and Torres Strait Islanders accessing community and government controlled AMS in Brisbane, Queensland with the intention of identifying self-perceived health determinants to inform the post-2015 international development goals.

**Methods:**

Focus group discussions and semi-structured interviews were held with clients of a government-controlled AMS and an Aboriginal community controlled health service (ACCHS) in order to identify their self-identified essential health needs. Conversations were audio recorded, transcribed verbatim and de-identified for analysis. Common themes were identified to highlight important issues around community health needs, how they can be addressed and what lessons can be extended to inform the post-2015 development goals.

**Findings and discussion:**

Participants acknowledge the complexity of health determinants faced by their peoples. Thematic analysis highlighted the pervasive influence of racism through many perceived health determinants; resulting in reduced healthcare seeking behaviour, unhealthy lifestyles and mental health issues. Participants emphasised the marked health improvements seen due to the establishment of Aboriginal medical services in their communities and the importance of the AMS’ role in addressing the negative effects of discrimination on Indigenous health.

**Conclusion:**

It is concluded from this study that AMS are crucial in addressing the negative impacts of continued discrimination on Indigenous health by providing comprehensive, culturally appropriate, community empowering health services. Such services improve Indigenous healthcare seeking rates, provide invaluable health education services and address mental health concerns in communities and must be supported in order to address health inequalities in Australia. Community driven and culturally informed health services should be encouraged globally to address health disparities.

## Introduction

Damning statistics expose Australia’s failure to improve third world health experienced by Aboriginal and Torres Strait Islanders in an advanced economy [1] ^a^. In 2009, the Australian Government recognised that Indigenous health, education and employment outcomes have not improved significantly in over a decade [2, 3]. In comparison to their non-Indigenous counterparts, Aboriginal and Torres Strait Islanders can expect to die almost 10 years earlier and experience a much higher burden of disease [4]; the Aboriginal infant mortality rate is almost twice that of non-Indigenous Australians and the child mortality rate is more than double [5]. Non-communicable diseases (NCDs) are more common [6], they are more likely to be fatal, and are more likely to be mistreated when presenting in Aboriginal people [7]. Aboriginal people are more than twice as likely to experience serious psychological distress, though definitive data is not readily available [8]. The gap between Indigenous and non-Indigenous wellbeing is similarly reflected in housing, water safety, and education resulting in a continuous cycle of ill-health and contributing determinants [9–13]. O’Sullivan states that “Equal access to good health is a mark of equal worth” and this persistent experience of lower standards of health highlights the institutionalised marginalisation of Aboriginal and Torres Strait Islanders in Australia [14].

The formation of the post-2015 sustainable development goals provides an opportunity for Australia to use the momentum of international advocacy for Universal Health Coverage, with its focus on equity, to improve healthcare standards for Aboriginal people and achieve equitable health for all. While the existing Millennium Development Goals (MDGs) are at the centre of the Australian foreign aid program [15], they have not been incorporated into domestic health goals. Australia’s health policy makers, along with those of most developed economies, have determined that these “global” goals to eradicate poverty, hunger, inequality and disease apply primarily to developing economies, despite obvious inequalities locally.

This research was conducted under the *Go4Health* project to identify self-perceived essential health needs of marginalised peoples around the world. Building on the existing MDGs, *Go4Health* seeks to shape the post-2015 development goals to increase the focus on equity both between countries but also within countries. *Go4Health* functions under the assumptions that people have the basic human right to participate in the decision making processes that will affect their lives and that if the new goals can meet the health needs of the most marginalised, they will meet the needs of all [16].

Indigenous peoples in developed economies have been identified as marginalised populations as they continue to experience a lower standard of health and socio-economic status in comparison to their respective non-Indigenous populations [1, 17]. While most developed, post-colonial countries have made impressive gains in Indigenous health in the last 20 years, little overall progress has been made in improving the health of Aboriginal and Torres Strait Islanders [13, 18]. As such, Indigenous Australians reflect the struggles faced by Indigenous populations in developed countries globally; and the health inequities and disparities experienced by marginalised population groups in high-income nations

This case study was undertaken in two different forms of urban Aboriginal medical service: one community-controlled health service; the second, State funded but headed by respected Aboriginal medical and nursing staff. Focus group discussions and one-on-one interviews with Aboriginal and Torres Strait Islanders were conducted to identify self-perceived community health determinants, how they can be addressed and whether their experiences can inform international health policy.

## Background

### History of colonisation and discrimination of aboriginal and Torres Strait Islanders

The marginalisation of Aboriginal and Torres Strait Islanders begins, as does that of most First Nations peoples, with colonisation; in this case by Great Britain from the late 18^th^ century [19]. The British did not recognise the existence of Aboriginals and employed the concept of *Terra nullius* - land belonging to no one - to justify their claim to their Australian colonies [20]. Despite early government intentions to deal fairly with those Indigenous peoples it encountered, abuse was common. Settlers kidnapped, abused and murdered locals, and destroyed sacred and economically essential environments in order to raise cattle and build settlements. Many Aboriginal groups were eradicated completely, and survivors were forced to squat and beg on the outskirts of British settlements, as settlement compromised their food sources and personal security [21].

Violence, introduced diseases, malnutrition, and substance abuse decimated Aboriginal communities, leading to the colonial belief that Aboriginal people were a dying race [19, 22]. ‘Protective’ legislation was implemented without Indigenous consultation and resulted in degrading, discriminatory and destructive policies. These included controlling marriages, movement, employment, and access to salaries, and placed many other restrictions on basic human rights; though did little to actually protect the people [22]. There was also forced relocation of Aboriginal people to poorly equipped Christian missions, Aboriginal reserves, or to stations for cheap labour. This relocation also served the purpose of segregating Aboriginal people from colonial society [23] and ‘controlling reproduction’; full-blooded Aboriginal people were corralled into missions and expected to die out. Children of mixed descent were often forcibly removed from their families, with the intent that they be ‘absorbed into civilised society’ in an effort to “breed out their blackness” [24].

In Queensland, the Industrial and Reformation Act of 1865 allowed the removal of ‘neglected’ children from their families to be placed in reformative schools. ‘Neglected’ legally included “any child born of an aboriginal or half-caste mother” [25]. Children were often removed by force, threat or coercion, and their treatment received in some schools has been likened to concentration camps [24]. Children were often emotionally, physically and sexually abused in schools and foster homes [24]. Similar laws were introduced across the country throughout the 19^th^ century and though implementation began haphazardly, by the 1930s the removal of children from their families became systemised [26]. So began an era of ‘assimilation’ whereby Aboriginal people were torn from their Country, banned from speaking their own languages, practising their own cultures or traditions, and children were removed from their families to be assimilated into mainstream society [20]. These children have been disadvantaged in every way; health, education, physique, psychological development and struggling to raise their own children due to a lack self-worth and parental guidance [24]. A survey conducted in 1994 found that more than 10% of Aboriginal people older than 25 years had been taken from their natural families and the trauma continues to deeply affect all Aboriginal people today [27].

### Indigenous concepts of health

Australia’s Aboriginal people are a diverse people with varying beliefs, cultures and languages. Therefore it is unsurprising that their understandings of health and health determinants are equally diverse and open to change [28]. Yet despite their heterogeneity, Indigenous Australians do share a more complex and holistic concept of health in comparison to the western biomedical model [29].

For Australian Aboriginal people there is no traditional word for “health” to denote a single, separate aspect of life [30]. Instead, Indigenous Australians subscribe to a number of concepts related to life balance that are deeply connected to family and society [31], to “Country” and way of life [32]. Health is spiritual, emotional, psychological, and physical and is deeply connected to the land of their ancestors, and to their past, present and future simultaneously [33]. As such, the health status of Aboriginal and Torres Strait Islanders cannot be isolated from their social, economic and historical state of colonisation, disconnection from their land and kin, and continuing marginalisation [34–37].

### Rise of Aboriginal Community Controlled Health Services (ACCHS)

Aboriginal Community Controlled Health Services (ACCHS) have developed to provide health care that caters specifically to the needs of Aboriginal clients, and are managed by the local Indigenous community through an elected board of directors. State controlled health facilities also provide health services for Aboriginal and Torres Strait Islander clients, with some specifically targeting Indigenous populations.

The first ACCHS was set up in 1971 in Sydney. For almost ten years, this service and all subsequent ACCHS were funded almost entirely by donations as state governments refused to provide support, arguing that Indigenous people had access to mainstream services [38]. While all AMS provided care for Indigenous Australians, ACCHS are distinct in that they are “culturally appropriate, autonomous primary health services initiated, planned and governed by local Aboriginal communities through their elected Aboriginal board of directors” [38]. They strive to achieve not just the physical health of an individual, but the “social, emotional and cultural wellbeing of a community within which an individual can achieve their full potential” [39] in accordance with Indigenous concepts of holistic health. This concept of health aligns strongly with the World Health Organisation’s definition of health as being “complete physical, mental and social well-being, and not merely the absence of disease and infirmity” [40], and should be Australia’s goal for all its citizens.

Government-controlled health services strove to meet the health needs of Indigenous Australians; however, although these services often employed Aboriginal health workers they rarely provided Aboriginal people with any real decision-making power until more recently. This minimised the efficacy of their ability to address community concerns [41]. The organisation, funding and responsibility of government-controlled services are fragmented between federal, state and local government, often resulting in delayed responses to community needs, inefficient provision of services and unreliable funding sources [42]. In contrast, ACCHS are each unique in their funding, management and community base, allowing rapid action in response to community needs [27].

Over a hundred community ACCHS currently exist in Australia. Funding through Medicare requires supplementation through short term program grants to maintain some services, causing problems for funding, staffing and sustainability, and leaving some ACCHS unable to provide comprehensive primary health care services to address their communities’ health needs [38]. The Australian Medical Association, and the Close the Gap campaign made up of peak Australian Indigenous and non-Indigenous health bodies, acknowledges that such services are the quintessence of comprehensive primary health care and must be supported in order to improve Indigenous health [43, 44].

## Methods

This research was approved by The School of Population Health Research Ethics Committee, the Institute of Urban Indigenous Health and the relevant Aboriginal community boards of participating groups.

Two focus group discussions were held with clients of two urban Indigenous health services in Brisbane; an ACCHS (n = 11) and a Queensland Government-controlled Aboriginal Health Service (n = 10). Access to the ACCHS groups was provided through the Institute of Urban Indigenous Health which leads Aboriginal health service planning, development and delivery in South East Queensland. Individuals from the discussion groups were then invited to participate in semi-structured one-on-one interviews. Eight participants from the ACCHS focus group provided individual interviews, with a further three who had not been present for the focus group discussion; three participants from the state Aboriginal Health Service provided in depth interviews. All participants identify as Aboriginal or Torres Strait Islanders, except for one participant of Cook Islander descent who accessed the ACCHS as a culturally appropriate alternative to mainstream services. Conversations were recorded with the oral consent of each participant and later transcribed verbatim and de-identified for analysis.

Focus group discussions and semi-structured in-depth interviews enabled the participants greater latitude to guide the themes of the study and highlight issues they considered to be of highest importance. The opportunity for participants to shape the discussions also ensured that sensitive issues raised were given respectful consideration and participants felt comfortable to speak freely.

Thematic analysis of transcribed data was conducted using NVIVO. Data was coded according to the five determinants of enquiry used to guide discussions across *Go4Health* consultations (community understandings of health, determinants of health, essential health needs and their provision, roles and responsibilities of relevant actors and community participation in decision making). Common themes were identified to highlight important issues around community specific essential health needs, how they can be addressed and what relevance their experiences might have to the international community.

This study is constrained by the limited numbers and single geographic location and cannot represent the views of all Indigenous Australians. However, as a case study, it provides a vignette of lay perspectives of Aboriginal and Torres Strait Islanders in an urban community of their experience of their health services and what they consider to be of significant importance to their health.

## Findings and discussion

The determinants of Aboriginal health, and indeed all health, are diverse and complex; and research participants cited a variety of needs as essential to addressing the disparity between Indigenous and non-Indigenous health experienced in Australia. These range from education and cultural identity to a lack of coherence in governance structures. Thematic analysis identified that historical and continuing racism permeated through major perceived determinants of Aboriginal health and participants’ beliefs that racism must be addressed in order to achieve true progress. Participants identified three major ways through which discrimination continues to affect Indigenous health: discrimination negatively impacts on health care seeking behaviour; it affects how Indigenous clients gain essential knowledge for their health and how it is passed on; and it directly affects the mental health of Australia’s first people. Despite this discouraging outlook, participants of this study spoke very highly of the health services they accessed, and how these issues are addressed through the provision of culturally appropriate and evidence-based services. This reflects widespread support for ACCHS and targeted health services as key to closing the gap between Indigenous and non-Indigenous health in Australia [45].

### Discrimination affecting healthcare seeking behaviour

Despite attending health services specifically for Aboriginal people, study participants all spoke of their hesitation to initially seek healthcare and of general apprehension within the community. While cost and accessibility were identified as barriers, participants emphasized how discomfort in mainstream health services results in delayed healthcare seeking. Participants highlighted three key factors that contribute to this discomfort as follow.

#### Mainstream clinics fail to acknowledge Indigenous concepts of health

Participants expressed concerns that mainstream clinics do not acknowledge or allow for Indigenous concepts of health. This often led to patient discomfort and miscommunication between patient and practitioner.

One participant speaks of barriers she found when working to introduce holistic and culturally appropriate health care practices in a mainstream general practice (GP) service in an area with a high population of Aboriginal people.

*“People* (in the mainstream GP service) *were not understanding the whole philosophy behind why this was needed. And that was for a number of reasons; one, people were just ignorant… they just did not want to know, they just wanted their money and that was it. And then you had other people who did understand it absolutely, but were not supported by their organisation to be able to do it.”* ACCHS administrator, female

The lack of culturally appropriate health care services remains a barrier to Aboriginal people accessing healthcare, but the successful integration of cultural safety principles-recognising the practitioners’ own cultural biases, and consciously engaging those of their clients- into mainstream health care results in dramatic increases in Aboriginal clientele. The participant speaks about the changes seen in that same GP service:

*“Once we imparted that skill or that knowledge to that particular general practice, and honestly, they had four Indigenous patients when we walked in and they had 450 when we walked out, 450 people when we walked out.”*

Mainstream clinics typically work reactively to treat symptoms but often fail to provide the support and information required to manage a disease in its cultural context; participants acknowledged this method of treatment left them feeling scared, confused and alone. Many Aboriginal health services, however, as part of the holistic and culturally appropriate heath care, provide support and preventative programs for clients. If diagnosed or identified as being ‘at risk’ of developing an NCD, clients are referred to support systems and allied health professionals on site to give them the tools, knowledge and support they need in order to manage or prevent the onset of a disease. A client contrasts the experience of her diabetes diagnosis in a mainstream GP clinic with the management of her mother in an ACCHS:

*“When I got diagnosed, that was it, ‘Off you go’… I walked out stunned, shocked, and thinking ‘Whoa, what does this mean? Am I going to die?’… And I said to him ‘What do I do now?’ and he goes, ‘Well, time’s up. Make another appointment.’… When* (my mother) *got diagnosed, right here in this clinic* (ACCHS)*, the doctor went and put her on a plan, she got diabetes educated, the dietician… She got diagnosed here and they’ve done everything for her here. This is what they need in communities.”* ACCHs client, female

The provision of preventative programs, support networks and management plans for clients who are diagnosed with NCDs empowers patients to take control of their situation and their lives [46]. Support groups also ensure that clients understand that they are not alone and provide safe places to ask questions and learn from one another. Participants expressed their hesitation to ask the doctor questions about their conditions:

*“Some people say ‘If there are any questions you’ve got to ask.’ But a lot of us don’t know what to ask and how to ask… So if you don’t know what to ask, you won’t find out. You never know what to ask.”* ACCHS client, female

Clients who are uncomfortable with a health professional or with their health condition are often unlikely to discuss their concerns but simply will not comply with lifestyle, treatment or medication recommendations; for a variety of cultural reasons, this is especially true for Indigenous Australians [47]. Support systems and educational groups initiated in a culturally appropriate way and in an emotionally safe environment encourage lifestyle changes that may save lives.

*“Having everybody turn up, we all know each other now… I’ve learnt more here than anywhere I’ve read or what the doctor can actually spare time for.”* ACCHS client, female

#### Fear of medical services

Negative inter-generational experience was a powerful continuing influence on Aboriginal families and their engagement with the broader health system. Study participants often drew on the negative health experiences experienced within their families to explain why people avoid healthcare services;

*“My mother-in-law, her mother took her brother into the doctors and he had some sort of disease. I don’t know what it was and they did something really radical. They amputated his leg because it was in his leg. And after that she never took any of the kids… A couple other of my mother-in-law’s brothers and sister died of illnesses that could have been prevented but because of that one problem… they’re scared of what’s going to happen.”* ACCHS client, female

The creation of health services that are initiated, planned and governed by local Aboriginal communities has provided a culturally safe environment that allows Aboriginal people to feel comfortable and welcome. Research participants described their local health service as “a healing centre”, and “a family”; this comfort is essential to encouraging Aboriginal clientele to access these services [48].

The negative association made with health services, though they may derive from experiences many years ago, creates a self-perpetuating cycle of fear, delay and worsening health conditions.

*“She said ‘I hate* [that] *hospital.’ … she said ‘Everyone I know who goes into* [that hospital] *dies.’ And a lot of people, because they don’t go early enough, they associate hospitals and going to the doctor with getting bad news and, not long after, dying.”* ACCHS client, Female elder

Having experienced comprehensive, culturally appropriate healthcare at an Aboriginal health service, study participants emphasised the need to reach out to the community and change the negative associations surrounding healthcare services.

*“They’re scared of what’s going to happen. But now it’s much better, but you’ve got to get the word out there to say that it’s not radical any more. It’s more trying to find a prevention than a cure.”* ACCHS client, female

In order to address this negative association, services for Aboriginal clients increasingly incorporate health awareness campaigns, education programs and community outreach into the services that they provide. Preventative measures are taken to diagnose NCDs and identify ‘at risk’ individuals by screening all Indigenous patients on first arrival at the service and regularly thereafter, and running community events with mandatory testing prior to individual participation.

#### Discrimination from health staff and other clients

Several participants stated that experiences of discrimination, or perceived discrimination, from staff and other clients at health facilities heavily influenced their attitude toward seeking healthcare.

*“I know that I should go to the doctor, and you just put it off, put it off, put it off, put it off, until it just escalates… and then you are in that situation where you have to go. And then you get there and you get – then you get – the racist remarks or comments.” ACCHS client, female*

ACCHS are designed to provide culturally appropriate health care, with culturally trained and Aboriginal staff to eliminate discrimination within services and ensure that clients feel comfortable when they seek health care [27, 38, 39, 45, 48]. Study participants voiced the importance of such treatment in their choice to seek healthcare at a culturally sensitive provider.

*“I was going to a doctor in Cleveland, and I didn’t feel comfortable there, but being here, where there’s other Aboriginal people around, yeah I felt so comfortable when I came here the first time… there were Aboriginal nurses as well… and you could relate to them a bit more. As if you’re talking to your own daughters or sisters.”* ACCHS client, Male elder

*“My Aunty and I had an argument because she said it’d be easier for me to go to the local doctors but I said, ‘No, I want to go to* [the AMS]*… I’m comfortable there.”* State Aboriginal Health Service client, female

*“He’s really good.* (The doctor) *is like that. I was ashamed to take my top off, and he said ‘You don’t have to.’ So* (the doctor) *is like that, good that he understands our cultural beliefs and that. I don’t feel uncomfortable now.”* ACCHS client, Female elder

### History of discrimination and disempowerment has damaged the passage of essential life skills

In traditional Aboriginal culture, knowledge is orally passed from elders to the young in the ritualised, respectful process of *storytelling* that has been maintained for thousands of years. Through this method of transmission, young people were taught how to survive in the bush and in society when they were deemed ready by their community elders [20]. Participants reflected on how colonisation resulted in the destruction of this passage of information. For example, the introduction of high sugar, high fat and highly processed foods combined with the removal of children from parents and elders has resulted in generations of Aboriginal people having been raised without knowledge of healthy lifestyles while simultaneously adjusting to Western influences.

*“I learnt a lot just being in the bush. Just being there and the old people, they taught you a lot of stuff. But the bush is gone, the old people are gone; we don’t have it no more.”* State Aboriginal Health ServiceMS client, male elder

*“We have never, ever been told the proper way to eat the right type of vegetables and how to cook properly… With my mum, they took her out of school, at the missions they were only allowed to go to grade four and then they had to go to work somewhere… and her mum wasn’t there to teach them… I think there was a generation that didn’t get educated enough about… what’s good for you and what’s not good for you.”* ACCHS client, male elder

Raising community awareness around healthy lifestyles is vital to addressing high rates of NCDs among Aboriginal people; however, participants emphasised the importance of raising awareness in a culturally appropriate manner, by paying respect to the elders and the traditional methods of *storytelling*. Participants acknowledged the efforts of schools attempting to encourage healthy eating at home but found it disrespectful and disempowering when children refused food that was offered to them by parents and elders. A male elder emphasised the need for Aboriginal elders to take control and revive the tradition of passing knowledge down, but updating the information to include healthy eating, exercise, child-raising, budgeting and other modern life skills. This effort must come from within the communities themselves, but an Aboriginal health service can provide support to initiate and maintain such programs within a community.

### Racism affecting mental health

Aboriginal people are twice as likely as non-Indigenous Australians to experience high or very high levels of psychological stress [49] which has been linked to reduced life expectancy and higher burden of disease [50]. Mental health contributes to 15% of the burden of disease for Aboriginal people, second only to cardiovascular disease [51].

There are many complex determinants behind the appalling mental health indicators for Aboriginal people, but participants of this study strongly felt that every-day racism was a major cause for mental health issues, and by extension, other health conditions.

*“I think we make each other sick. That is what I believe. And then we end up with cancer, we end up with diabetes, we end up with all these other diseases, and I think because of social hatred… Because when you are combative like we are, on a day-to-day basis of racism, of not getting served when we know that we are in line and you just let it go, and you just stand there with a smile on your face… So you see the psychological abuse that you get? You are just constantly told that you are no good – not good enough.”* ACCHS client, female

Increasingly, mental health is being addressed in health services with the provision of psychiatric consultations and support groups; however, institutionalised and day-to-day racism that is affecting the health of Aboriginal and Torres Strait Islanders cannot be addressed through the health sector alone. Addressing this issue requires a multi-sectoral approach to change the perceptions and behaviour of all Australians.

*“You think racism is dead? It is alive and it is thriving out there and people have got unwritten permission to impact on Aboriginal people.”* ACCHS client, female

*“I’ve come to this horrible, negative space, but coming here* [ACCHS] *is helping me get back… it’s like, for me coming to this program is like I’m on a journey, a journey of healing for me, yeah, to get healthy, stronger and live longer.”* ACCHS client, female

## Discussion

Discrimination has been well documented as impacting negatively on health; both directly, through violence and affecting mental health, and indirectly, by minimising access to essential services such as healthcare, education and infrastructure [52–56]. Many studies have highlighted the importance of culturally appropriate, community-controlled, comprehensive health services to maximise health benefits to a community [27, 36, 38, 56]. This study strengthens the evidence that racism, historical and current, continues to restrict the improvement of Indigenous health in Australia and the existence of culturally safe and comprehensive medical services is crucial to addressing these negative impacts by improving healthcare seeking behaviour, providing health education services, and addressing mental health issues in Aboriginal communities.

Several studies have found that if facilities are not culturally appropriate, they are less likely to be accessed by Aboriginal clients [33, 55–57]. Discomfort, cultural exclusion and fear are the main reasons Aboriginal people give for avoiding healthcare services; often avoiding care until they are seriously ill, which contributes to much lower survival rates for NCDs among Aboriginal people than non-Indigenous Australians [7, 33, 38]. A survey conducted in 2005 found that 15% of Indigenous Australians had needed to see a doctor in the previous 12 months but had not [58]. Distance, cost and family commitments also contribute to reduced healthcare seeking behaviour [31].

Since the 1980s, the Western understanding of effective health provision has shifted away from the purely biomedical model of health, which strips away the political, social, cultural and economic context that is vital to addressing health concerns at the source [36, 59, 60]. Western health models are recognising the more complex determinants of health and ill-health and are coming to understand that patients want to make good health decisions for themselves [46] and must be empowered with tools and information to do so [56]. This holistic concept of health corresponds to the Aboriginal understanding of health; however, mainstream Australian health care centres often still operate under the biomedical model with its assumption of medical compliance, where patients are expected to comply unquestioning with the doctor’s directions [61]. This continued refusal to incorporate Indigenous health perspectives into mainstream health care fails to address Indigenous health needs and can clash directly with what is culturally appropriate to Aboriginal people, working to persistently marginalise the Aboriginal population and discouraging them from accessing health care [62].

It is well acknowledged that programs that incorporate Aboriginal lay perspectives are far more successful in improving Indigenous health than those that ignore them [51, 63–65]. Incorporating Aboriginal voices and cultural perspectives into the design, implementation and evaluation of Indigenous health programs improves community health by developing effective, culturally appropriate intervention methods, by improving communication between clients and health care professionals and by increasing healthcare-seeking behaviour among the Aboriginal population [47, 51, 65, 66]. ACCHS are initiated, planned and governed by a board of directors chosen by the community they serve, ensuring cultural safety and community involvement at all times.

In addition to experiences and expectations of cultural insensitivities in mainstream healthcare centres, the broader history of marginalisation and systematic maltreatment at the hands of the authorities has understandably instigated a sense of distrust for western institutions,. and hesitance to seek care unless absolutely necessary [67–69]. As a result health problems are often far more advanced when treatment is sought, and risks of severe complications or dying are increased [6], creating an association of health centres with death and radical interventions. This creates a perpetual cycle of delayed care, increased death and burden of disease, fear of health services and then, delaying care.

There have been calls to incorporate *storytelling* into Indigenous health promotion strategies [70]. Although modern health promotion that incorporates traditional *storytelling* must be community-driven and culturally-appropriate, health services can support and facilitate such services through funding, advertising, providing a client base or providing a place where people can come together.

Health services for Aboriginal and Torres Strait Islander populations also provide holistic, comprehensive and culturally appropriate care by working with the greater community to prevent rather than cure, and educate people about healthier life choices. ACCHS offer a broader political empowerment to communities; as they are community driven, granting local people the ability to take control of their own health and the services that are provided to them. Every inquiry conducted in the last 30 years has acknowledged ACCHS as crucial to closing the gap between Indigenous and non-Indigenous health in Australia [43–45].

## Conclusion

There are no easy solutions when addressing a national, racial health disparity as ingrained as that between Indigenous and non-Indigenous Australians. The determinants of ill-health are complex and the social inequity faced by Aboriginal people cannot be solved by the provision of biomedical primary health care alone. ACCHS use culturally informed, community empowering, preventative, supportive and primary health care services to address the downstream effects of this inequality and racism and are improving health in the communities they serve.

*“My mum and dad would be here now if they would have had the medical things like this in their time.”* ACCHS client, Male elder

However, these services are an underfunded, understaffed and incomplete response to the historical and continued marginalisation of Aboriginal and Torres Strait Islanders. For Australia to achieve health for all, there must be political action to improve services in a multi-sectoral approach; including housing, education, health awareness, community empowerment, infrastructure, governance of Indigenous issues as well as support for Aboriginal health services. The presence of racism as a determinant of health must be officially recognised and addressed through knowledge and culture sharing. Indigenous health must be promoted to higher importance in Australia’s post-2015 domestic in-country targets and the momentum of the post-2015 sustainable development goals provides the perfect opportunity for Australia to do so.

## Endnote

^a^In this paper, ‘Aboriginal’ and ‘Indigenous Australians’ refers to Aboriginal and Torres Strait Islander people as the first and Indigenous inhabitants of Australia [2].
